# Circulating and Synovial Pentraxin-3 (PTX3) Expression Levels Correlate With Rheumatoid Arthritis Severity and Tissue Infiltration Independently of Conventional Treatments Response

**DOI:** 10.3389/fimmu.2021.686795

**Published:** 2021-06-25

**Authors:** Marie-Astrid Boutet, Alessandra Nerviani, Gloria Lliso-Ribera, Roberto Leone, Marina Sironi, Rebecca Hands, Felice Rivellese, Annalisa Del Prete, Katriona Goldmann, Myles J. Lewis, Alberto Mantovani, Barbara Bottazzi, Costantino Pitzalis

**Affiliations:** ^1^ Centre for Experimental Medicine & Rheumatology, William Harvey Research Institute and Barts and The London School of Medicine and Dentistry, Queen Mary University of London, London, United Kingdom; ^2^ Inserm UMR 1229, Regenerative Medicine and Skeleton, RMeS, Université de Nantes, ONIRIS, Nantes, France; ^3^ Department of Inflammation and Immunology, Humanitas Clinical and Research Center—IRCCS, Milan, Italy; ^4^ Department of Molecular and Translational Medicine, University of Brescia, Brescia, Italy; ^5^ Department of Biomedical Sciences, Humanitas University, Milan, Italy

**Keywords:** pentraxin-3, rheumatoid arthritis, synovial tissue, pathotypes, inflammation

## Abstract

**Aims:**

To determine the relationship between PTX3 systemic and synovial levels and the clinical features of rheumatoid arthritis (RA) in a cohort of early, treatment naïve patients and to explore the relevance of PTX3 expression in predicting response to conventional-synthetic (cs) Disease-Modifying-Anti-Rheumatic-Drugs (DMARDs) treatment.

**Methods:**

PTX3 expression was analyzed in 119 baseline serum samples from early naïve RA patients, 95 paired samples obtained 6-months following the initiation of cs-DMARDs treatment and 43 healthy donors. RNA-sequencing analysis and immunohistochemistry for PTX3 were performed on a subpopulation of 79 and 58 synovial samples, respectively, to assess PTX3 gene and protein expression. Immunofluorescence staining was performed to characterize PTX3 expressing cells within the synovium.

**Results:**

Circulating levels of PTX3 were significantly higher in early RA compared to healthy donors and correlated with disease activity at baseline and with the degree of structural damages at 12-months. Six-months after commencing cs-DMARDs, a high level of PTX3, proportional to the baseline value, was still detectable in the serum of patients, regardless of their response status. RNA-seq analysis confirmed that synovial transcript levels of PTX3 correlated with disease activity and the presence of mediators of inflammation, tissue remodeling and bone destruction at baseline. PTX3 expression in the synovium was strongly linked to the degree of immune cell infiltration, the presence of ectopic lymphoid structures and seropositivity for autoantibodies. Accordingly, PTX3 was found to be expressed by numerous synovial cell types such as plasma cells, fibroblasts, vascular and lymphatic endothelial cells, macrophages, and neutrophils. The percentage of PTX3-positive synovial cells, although significantly reduced at 6-months post-treatment as a result of global decreased cellularity, was similar in cs-DMARDs responders and non-responders.

**Conclusion:**

This study demonstrates that, early in the disease and prior to treatment modification, the level of circulating PTX3 is a reliable marker of RA activity and predicts a high degree of structural damages at 12-months. In the joint, PTX3 associates with immune cell infiltration and the presence of ectopic lymphoid structures. High synovial and peripheral blood levels of PTX3 are associated with chronic inflammation characteristic of RA. Additional studies to determine the mechanistic link are required.

## Introduction

Rheumatoid arthritis (RA) is the most common chronic inflammatory autoimmune joint disease. It is characterized by synovitis and progressive cartilage destruction and bone erosions, and can lead to persistent joint pain and functional disability. Defective control of the systemic inflammatory response and the development of autoimmunity, often emerging long before the clinical onset of the disease, result in the synovial infiltration and activation of immune and stromal cells that lead to the production of high levels of inflammatory mediators such as pro-inflammatory interleukins (IL-1, Tumor Necrosis Factor-TNF-α, IL-6) or matrix metalloproteinases (MMP) (1). These molecules contribute to the maintenance of synovitis, which displays histopathological variability that has been categorized into three partially overlapping pathotypes (2). These include the lympho-myeloid pathotype characterized by the presence of B/T cells forming organized ectopic lymphoid structures (ELS), plasma cells and macrophages; the diffuse-myeloid pathotype with a predominant infiltration of macrophages without organized ELS, and the pauci-immune fibroid pathotype defined by a scant immune cell infiltrate. These pathotypes have been shown to stratify disease activity and severity, as well as response to conventional synthetic (cs) disease modifying anti-rheumatic drug (DMARDs) (3). Furthermore, soluble proteins such as the classical short pentraxin C-reactive protein (CRP), in addition to acting as acute phase reactants and indicator of systemic inflammation, also reflect joint disease activity and tissue pathology (3, 4).

The long pentraxin 3 (PTX3) is a soluble pattern recognition molecule highly conserved in evolution (5). PTX3 is generally poorly expressed in homeostatic conditions, but is rapidly upregulated in response to inflammatory stimuli. For instance, the proinflammatory molecules IL-1 and TNF are the most important inducers of PTX3 production by myeloid and endothelial cells. When appropriately stimulated, fibroblasts and other stromal cells also release PTX3. PTX3 is a multifunctional protein exerting non-redundant roles in the innate resistance to pathogens, the regulation of inflammation and tissue repair. It has been shown that PTX3 can limit tissue damage in murine models of acute lung injury and ischemia/reperfusion-induced kidney injury by regulating neutrophil recruitment at inflamed sites (6, 7). In experimental models of chemically-induced sterile liver and lung injury, skin wound healing and in arterial thrombosis, PTX3-deficiency is associated with altered remodeling of the fibrin-rich inflammatory matrix and impairment of normal tissue repair (8). Similarly to CRP, PTX3 circulating levels are increased in several inflammatory or infectious pathological conditions and are associated with disease severity and mortality risk (9–11).

In the context of RA, PTX3 has been shown to be constitutively highly expressed by synovial fibroblasts (12); however, as described in other tissues, various resident or infiltrating synovial cells like macrophages or endothelial cells might also represent a source of PTX3 upon inflammatory activation. Several groups found that PTX3 is upregulated in the serum of RA patients compared to healthy donors (13–15) or patients with osteoarthritis (16). However, conflicting results have been published regarding the relationship between the level of PTX3 in the serum and clinical features of the disease. On the one hand, studies on different cohorts of 111 (17), 29 (18), 60 (13), 83 (19), 58 (14) or 41 (15) RA patients reported no relationship between PTX3 and the Disease Activity Score (DAS)-28. Conversely, other groups found an association between PTX3 serum levels and either CRP (16) or the progression of joint damage assessed by increase in the total van der Heijde modified Sharp score (SHSS) and joint space narrowing over 3 years (20). In addition, Weitoft and colleagues reported that the level of PTX3 in synovial fluid from RA patients is higher in seropositive patients (anti-cyclic citrullinated protein -CCP- antibody or rheumatoid factor -RF-) when compared to seronegative patients, and correlates with disease activity and local expression of markers of inflammation such as IL-6 or vascular endothelial growth factor (VEGF) (21). These contradictory results likely depend on the heterogeneity of the cohorts analyzed including, for example, diverse disease stages, treatment exposure (cs- or biologic-DMARDs, known to influence various biologic processes), and disease phases (acute flare/latent chronic). For example, during acute flares, a dysregulated production of synovial fluid with a predominant infiltration of neutrophils will significantly influence PTX3 level.

Here, to clarify the relationship between PTX3 systemic and synovial levels and clinical parameters while minimizing population heterogeneity and drug-exposure, we investigated a large cohort of treatment-naïve early RA patients. We performed a thorough analysis of PTX3 circulating and transcript/protein synovial expression at baseline and 6-months after cs-DMARDs exposure in matched individuals. Here, we provide important insights into the relevance of PTX3 in RA disease tissue pathology, disease severity and whether it is a useful biomarker predictive of treatment response in RA.

## Materials and Methods

### Patients

Blood samples and synovial tissues were collected from early (<12 months of symptoms) treatment-naïve RA patients enrolled into the Pathobiology of Early Arthritis Cohort (PEAC) at Bart’s Health NHS Trust (http://www.peac-mrc.mds.qmul.ac.uk/). All RA patients fulfilled the 2010 American College of Rheumatology/European League Against Rheumatism (ACR/EULAR) criteria (22). The study was approved by the National Research Ethics Service Committee London Dulwich (REC 05/Q0703/198). A signed informed consent was obtained by all patients enrolled in the study.

At baseline, patients underwent ultrasound-guided needle synovial biopsy of an actively inflamed joint, as previously described (23), before commencing cs-DMARDs treatment. Synovial tissues were embedded in paraffin for histological characterization or preserved in RNA later (Ambion, Invitrogen, Carlsbad, CA, USA) for gene expression analysis. Sera were obtained by centrifugation of blood at room temperature for 10 minutes, aliquoted and stored at -80°C for further use. Six-months after starting treatment, patients were consented to undergo a repeated synovial biopsy of the same joint as baseline and a post-treatment serum sample was also collected.

At both baseline and 6-months, number of swollen and tender joints, patient visual analogue score (VAS) for pain, fatigue, global health, and physician assessment of global health were recorded. Erythrocyte sedimentation rate (ESR) and CRP were also measured. Rheumatoid factor (RF) and anti-cyclic citrullinated peptide (CCP) antibodies in the serum were assessed and recorded at baseline, as per the RA 2010 classification criteria (22).

The clinical response to cs-DMARDs was assessed according to EULAR criteria based on the achieved DAS28 and the difference between baseline/post-treatment DAS28. Accordingly, patients were classified as non-, moderate- or good-responders (24).

Structural damage was assessed at baseline and at 12-month follow-up by scoring hand and feet radiographs according to the SHSS performed by a single reader blinded to all clinical/histological data.

Control serum samples were collected from 43 age- and gender-matched healthy volunteers at Humanitas Research Hospital.

### RNA Sequencing (RNA-Seq)

Total RNA was extracted from RA synovial tissues using a Trizol/Chloroform method. Bulk RNA-seq was performed on an Illumina HiSeq2500 platform (Illumina Inc., San Diego, CA, USA). Raw data quality control, normalization and analysis of regularized log expression read counts were performed as previously described (4). RNA-seq data were uploaded to ArrayExpress and are accessible *via* accession E-MTAB-6141. Gene set enrichment analysis (GSEA) was performed using the R interface to the EnrichR database (https://CRAN.R-project.org/package=enrichR). The PTX3 gene module was composed of the 30 genes encoding for the protein integrated in the String network for PTX3 (25).

### Enzyme-Linked Immunosorbent Assay (ELISA)

PTX3 levels were measured in serum samples from healthy donors (n=43) and RA patients (119 baseline and 95 follow-up samples) as previously described (26), using a sandwich ELISA assay developed in-house (detection limit 0.1 ng/mL, inter-assay variability from 8% to 10%), by staff blinded to patients’ characteristics. All samples were run in technical duplicates.

### Histology, Immunohistochemistry (IHC) and Immunofluorescence (IF)

Sections (3 μm thick) of synovial tissue were stained by IHC using antibodies specific for B cells (CD20, Dako, Agilent Technologies, Santa Clara, CA, USA), T cells (CD3, Dako), plasma cells (CD138, Dako) or macrophages (CD68, Dako) to determine their degree of cellular infiltration. Synovial samples were categorized into three pathotypes (pauci-immune, diffuse myeloid or lympho-myeloid) following semi-quantitative scoring by two independent observers (2, 3). Briefly, the lympho-myeloid pathotype is characterized by the presence of B/T cells frequently forming highly-organized ectopic lymphoid structures (ELS), plasma cells and abundant macrophages in the sublining; the diffuse-myeloid pathotype is marked by a predominant infiltration of CD68+ macrophages without distinctly organized follicular structures; and the pauci-immune pathotype is defined by a scant immune cells infiltrate and a fibroblast-rich stroma. Samples were classified as ungraded if there was no recognizable synovial tissue or if the tissue was of insufficient quality.

Synovial tissues were also stained for PTX3 [affinity purified rabbit IgG anti-human PTX3, generated in-house - (27)]. Matching isotype controls were used to confirm the specificity of the primary antibodies. Slides were counterstained with hematoxylin and mounted with Distyrene Plasticizer Xylene (DPX) mounting medium (Sigma-Aldrich, Saint-Louis, MO, USA). All sections were digitally scanned using Nanozoomer S210 (Hamamatsu Photonics, Japan). Quantitative digital image analyses were performed to determine the percentage of PTX3-positive cells within the synovial tissue using QuPath software (28).

Double fluorescent labeling was performed on synovial sections by multiplex immunofluorescence staining using a tyramide signal amplification protocol (Invitrogen, Thermo Fisher Scientific, Waltham, MA, USA). PTX3 expression was evaluated in combination with the expression of the following markers: CD68, CD138, CD55 (Thermo Fisher Scientific), TE-7 (Merck, Darmstadt, Germany), lymphatic vessel endothelial hyaluronan receptor 1 (LYVE-1, Abcam, Cambridge, UK), von Willebrand factor (vWF, Dako) and Neutrophil Elastase (NE, Novus Biologicals, Centennial, CO, USA). Slides were counterstained with 40,6-diamidino-2-phenylindole (DAPI) (Invitrogen, Thermo Fisher Scientific) and mounted with ProLong Antifade Mountant (Thermo Fisher Scientific). Sections were digitally scanned using Nanozoomer S60 (Hamamatsu Photonics).

### Statistical Analysis

Differences in continuous variables were evaluated by the Mann Whitney U test (unpaired samples, two groups, <30) or Wilcoxon test (paired samples, two groups, <30), unpaired or paired Student’s t test (two groups, >30) or Kruskal Wallis with Dunn’s post-test (multiple groups). Chi-squared/Fisher’s exact test were used to evaluate associations of categorical variables. Correlations were evaluated by Spearman’s bivariate analysis. Statistical analyses were performed using GraphPad Prism-v9 software (Graphpad, San Diego, CA, USA). p-values <0.05 were considered significant.

## Results

### Baseline and 6-Months Patients’ Characteristics

Baseline demographics and clinical features of the 119 RA patients included in this study are summarized in **Table 1**. As expected for a cohort of RA patients, the female-to-male ratio was approximately 3:1 (68.9% female), the median age was 51.0 and 67.2% of the patients were positive for either RF or anti-CCP autoantibodies. As per the inclusion criteria, patients had early RA with a median disease duration of 5.3 months and all patients had active joint disease (tender joint count/28 11/28; swollen joint count/28 6/28; DAS28 5.9). At baseline, 38.7% of the synovial tissues were classified as lympho-myeloid, 28.6% as diffuse-myeloid, 21% as pauci-immune and 11.8% were ungraded. As previously reported by our group (3), patients presenting a lympho-myeloid pathotype had significantly more active disease, with higher ESR and CRP levels and DAS28 scores. In addition, patients with a lympho-myeloid pathotype were more likely to be seropositive for RF or anti-CCP, though the result did not reach statistical significance.

**Table 1:** Patient’s characteristics at baseline and at 6-months after treatment.

		Total population n = 119	Lympho-myeloid n = 46	Diffuse-myeloid n = 34	Pauci-immune n = 25	p value
**BASELINE**	**Female** % (n)	68.9% (82)	73.9% (34)	70.6% (24)	68.0% (17)	0.86, ns
	**Age** years, median (IQR)	51.0 (42.5;64.0)	55.5 (45.5;61.8)	49.0 (37.3;65.8)	51.0 (42.0;64.0)	0.47, ns
	**Disease duration** months, median (IQR)	5.3 (3.0;8.0)	5.8 (3.0;8.0)	4.5 (3.0;7.0)	5.0 (3.0;7.3)	0.75, ns
	**RF+** % (n)	65,5% (78)	78.3% (36)	67.6% (23)	52.0% (13)	0.07, ns
	**Anti-CCP+** % (n)	57.1% (68)	71.7% (33)	55.9% (19)	44.0% (11)	0.06, ns
	**RF+ or anti-CCP+** % (n)	67.2% (80)	80.4% (37)	67.6% (23)	56.0% (14)	0.19, ns
	**ESR** mm/hr, median (IQR)	36.0 (19.0;53.0)	52.0 (35.5;72.5)	29.0 (19.0;44.0)	19.0 (11.0;38.0)	0.0001, ***
	**CRP** mg/l, median (IQR)	9.0 (5.0;22.0)	20.0 (7.0;40.0)	9.0 (6.0;18.0)	5.0 (5.0;7.0)	0.0001, ***
	**PTX3** ng/mL, median (IQR)	3.2 (2.3;5.0)	3.6 (2.2;5.4)	3.7 (2.6;5.3)	3.1 (2.2;4.1)	0.48, ns
	**TJC/28**, median (IQR)	11.0 (6.0;16.0)	12.0 (9.3;15.0)	9.0 (4.3;12.8)	9.0 (5.0;19.0)	0.07, ns
	**SJC/28**, median (IQR)	6.0 (4.0;9.0)	8.0 (5.0;10.0)	6.0 (4.0;8.0)	5.0 (2.0;10.0)	0.06, ns
	**VAS fatigue**, median (IQR)	42.0 (15.0;64.0)	47.5 (13.5;68.5)	36.5 (14.3;63.8)	42.0 (22.0;56.0)	0.75, ns
	**VAS pain**, median (IQR)	56.0 (30.0;74.5)	61.5 (46.3;75.8)	47.0 (24.5;72.0)	49.0 (24.0;74.0)	0.13, ns
	**VAS global patient**, median (IQR)	72.0 (51.0;84.0)	76.5 (62.0;83.8)	71.0 (52.5;87.8)	68.0 (39.0;78.0)	0.40, ns
	**VAS global physician**, median (IQR)	66.0 (47.0;77.0)	72.0 (57.3;83.3)	55.5 (46.3;71.5)	48.0 (35.0;69.0)	0.002,**
	**HAQ**, median (IQR)	1.5 (1.0;2.0)	1.8 (1.2;2.1)	1.3 (1.0;2.1)	1.4 (0.9;2.0)	0.34, ns
	**DAS28**, median (IQR)	5.9 (5.0;6.8)	6.5 (5.8;7.0)	5.4 (4.9;6.2)	5.1 (4.4;6.5)	0.0008, ***
	**DMARDs treatments received** % (n)	94.1% (111)	97.8% (45)	97.1% (33)	84.0% (21)	0.04,*
	**Steroids treatments received** % (n)	47.9% (57)	65.2% (30)	44.1% (15)	36.0% (9)	0.04,*
		**Total population** n=95	**Lympho-myeloid** n=22	**Diffuse-myeloid** n=25	**Pauci-immune** n=37	**p value**
**6-MONTHS**	**ESR** mm/hr, median (IQR)	13.0 (7.0;31.0)	27.0 (9.0;34.0)	14.0 (8.0;29.0)	12.0 (4.0;31.0)	0.18, ns
	**CRP** mg/l, median (IQR)	5.0 (5.0;7.0)	5.0 (5.0;7.0)	5.0 (5.0;6.3)	5.0 (5.0;5.0)	0.98, ns
	**PTX3** ng/mL, median (IQR)	4.0 (2.7;5.2)	4.1 (5.5;5.1)	3.4 (2.8;4.5)	4.0 (2.9;5.8)	0.59, ns
	**TJC/28**, median (IQR)	3.0 (0;9.5)	4.0 (0.3;9.0)	3.0 (1.0;7.0)	3.0 (0;14.0)	0.93, ns
	**SJC/28**, median (IQR)	2.0 (0;4.0)	2.0 (1.0;5.8)	0.0 (0;3.0)	1.0 (0;3.0)	0.26, ns
	**VAS fatigue**, median (IQR)	33.5 (9.0;57.5)	23.5 (1.8;51.5)	34.0 (5.0;68.0)	41.5 (16.8;55.8)	0.35, ns
	**VAS pain**, median (IQR)	18.0 (4.0;64.0)	26.0 (4.8;70.5)	35.0 (4.0;75.0)	19.5 (3.0;51.3)	0.51, ns
	**VAS global patient**, median (IQR)	31.0 (5.5;68.5)	35.0 (8.3;69.5)	41.0 (4.0;71.0)	24.0 (7.0;67.0)	0.91, ns
	**VAS global physician**, median (IQR)	20.0 (4.3;47.3)	23.0 (16.0;38.5)	33.0 (5.0;55.0)	15.5 (3.0;38.3)	0.63, ns
	**HAQ**, median (IQR)	0.8 (0;1.7)	1.1 (0.4;1.6)	0.9 (0;2.0)	0.5 (0;1.8)	0.74, ns
	**DAS28**, median (IQR)	3.4 (2.0;5.3)	4.0 (2.7;5.3)	3.3 (2.5;5.0)	3.1 (1.7;6.3)	0.50, ns
	**Responders to DMARDs** % (n)	77,9% (74)	77.3% (17)	84.0% (21)	73.0% (27)	0.60, ns

The total population is represented in gray (n=119 at baseline and 95 at 6-months) and subdivided according to the synovial pathotypes (lympho-myeloid, diffuse-myeloid and pauci-immune), 14 synovial tissue at baseline and 11 at 6 months were ungraded (absence of lining layer and/or presence of necrotic tissue). p-values were calculated by comparing the values obtained for the three pathotypes groups using Kruskal Wallis or Fisher’s exact test as appropriate. n, number; IQR, interquartile range; RF, rheumatoid factor; CCP, cyclic citrullinated peptide; ESR, erythrocyte sedimentation rate; CRP, C-Reactive Protein; PTX3, pentraxin-3; TJC, tender joints count; SJC, swollen joints count; VAS, Visual Analogue Scale (0-100); HAQ, Health Assessment Questionnaire; DAS, Disease Activity Score; DMARDs, Disease Modifying Anti-Rheumatic Drugs; *p < 0.05; **p < 0.01; ***p < 0.001; ns, non-significant.

Following the baseline clinical assessment, 94.1% of the patients received cs-DMARDs and 47.9% received adjunctive corticosteroid treatment. Ninety-five patients (79.8% of the baseline population) successfully completed their 6-month follow-up visit and, at this timepoint, 77.9% of patients were classified as responders to treatment according to EULAR response criteria, with a similar response rate in the three pathotype groups.

### Levels of Circulating PTX3 Correlate With Disease Severity in Early Untreated RA Patients, but Not With Clinical Response to cs-DMARDs Treatment

**Figure 1:**
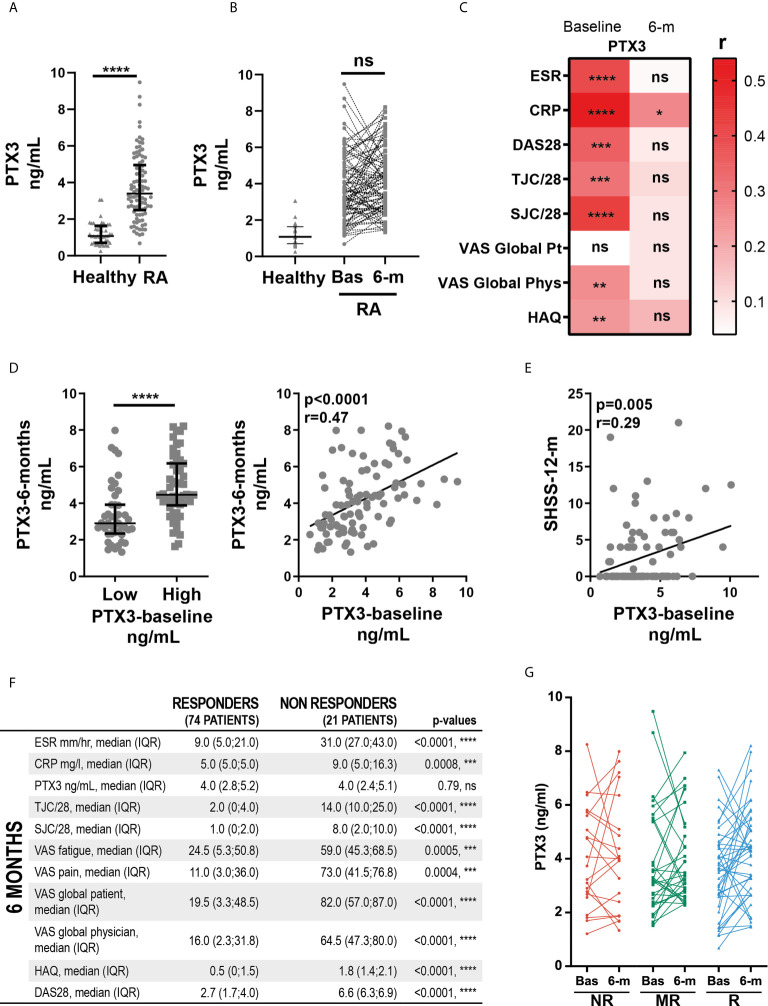
Levels of circulating PTX3 correlate with disease severity in early untreated RA patients, but not with clinical response to cs-DMARDs treatment. **(A)** PTX3 serum levels in patients with early treatment-naïve RA (n=119) and age- and gender-matched healthy donors (n=43). ****p < 0.0001, as assessed by parametric unpaired t-test. **(B)** PTX3 serum levels in healthy donors (n=43) and in paired patients at baseline (Bas) (n=119) and at 6-months (n = 95). ns, non-significant, as assessed by the parametric paired t-test comparing RA baseline and 6-months samples. **(C)** Left panel, PTX3 serum levels at 6-months in patients presenting a low (≤3.23 ng/mL) or high (>3.23 ng/mL) PTX3 serum levels at baseline, ****p < 0.0001 assessed by the parametric unpaired t-test. Right panel, correlation between PTX3 serum levels at baseline and at 6-months, p-value and r coefficient were calculated according to the Pearson correlation test. **(D)** Heatmap representing the correlation between PTX3 serum levels at baseline or 6-months (6-m) after cs-DMARDs treatment with clinical parameters: erythrocyte sedimentation rate (ESR), C-Reactive Protein (CRP), Disease Activity Score 28 (DAS28), Tender and swollen joint counts (TJC/28, SJC/28), patient (Pt) and physician (Phys) global health assessment by Visual Analogue Scale (VAS) and Health Assessment Questionnaire (HAQ). The red scale represents the spearman r coefficient. *p < 0.05, **p < 0.01, ***p < 0.001, ****p < 0.0001, as assessed by the Pearson correlation test. **(E)** Correlation between PTX3 serum levels at baseline and the van der Heijde modified Sharp score (SHSS) assessed at 12-months, p-value and r coefficient were calculated according to the Pearson correlation test. **(F)** Table presenting the clinical characteristics of responders (good and moderate) and non-responders patients to cs-DMARD treatments at 6-months, according to EULAR criteria. **(G)** PTX3 serum levels at baseline (Bas) or at 6-months (6-m) in non-responders (NR, red lines), moderate responders (MR, green lines) and good responders (R, blue lines) patients to cs-DMARD treatments. (**A–C** Left panel) Data are represented as median +/- interquartile range.

PTX3 was significantly higher in the serum of RA patients (n=119) in comparison with age- and gender-matched healthy donors (n=43), as expected in association with the inflammatory status of these patients (healthy donors median 1.25, IQR 0.76-1.64; RA median 3.23, IQR 2.28-4.99; **Figure 1A**). The overall PTX3 systemic levels were similar at baseline and at 6-months after treatment with cs-DMARDs (n= 94 paired samples) (**Figure 1B**). However, paired analysis revealed that patients with a high baseline PTX3 serum level (values higher than the median, i.e. >3.23 ng/mL) also had significantly higher circulating PTX3 at 6-months after starting treatment compared to the low-PTX3 (≤3.23 ng/mL) baseline patients group (**Figure 1C**, left panel); consistently, we detected a significant correlation between PTX3 levels at baseline and at 6-months (**Figure 1C**, right panel). We observed that serum PTX3 positively correlated with clinical parameters such as CRP, ESR, DAS28, tender and swollen joints, VAS physician assessment of global health and HAQ at baseline. However, for most of these variables, except for the CRP, the correlation was lost at 6-months after starting treatment (**Figure 1D**). Importantly, the baseline serum level of PTX3 also positively correlated with the degree of bone erosions assessed by the SHSS at 12-months (**Figure 1E**). However, a low/high clinical activity or erosion status at baseline did not influence the evolution of PTX3 levels between baseline and 6-months (**Supplementary Figure 1A**). Although all clinical variables assessed at 6-months were significantly reduced in patients who responded to treatment according to EULAR response criteria (**Figure 1F**), PTX3 expression was not affected by cs-DMARDs, and both PTX3 concentrations at each timepoint and the delta-PTX3 between baseline and 6-months were similar in all patients, regardless of their response status (none, moderate or good responders) (**Figure 1G** and **Supplementary Figure 1B**). Moreover, there was no difference between patients treated with different cs-DMARDs or who received steroids (*data not shown*).

Next, we analyzed the expression and distribution of PTX3 in the synovial tissue of a subset of patients both at the gene (n=79) and protein (n=58) level by RNA-seq and IHC, respectively. As shown in **Supplementary Table 1**, these subgroups of patients displayed similar demographic and clinical characteristics as the total population (n=119) above in which we analyzed systemic serum levels.

### Synovial PTX3 Gene Expression Correlates With Disease Activity and Local Inflammation

**Figure 2:**
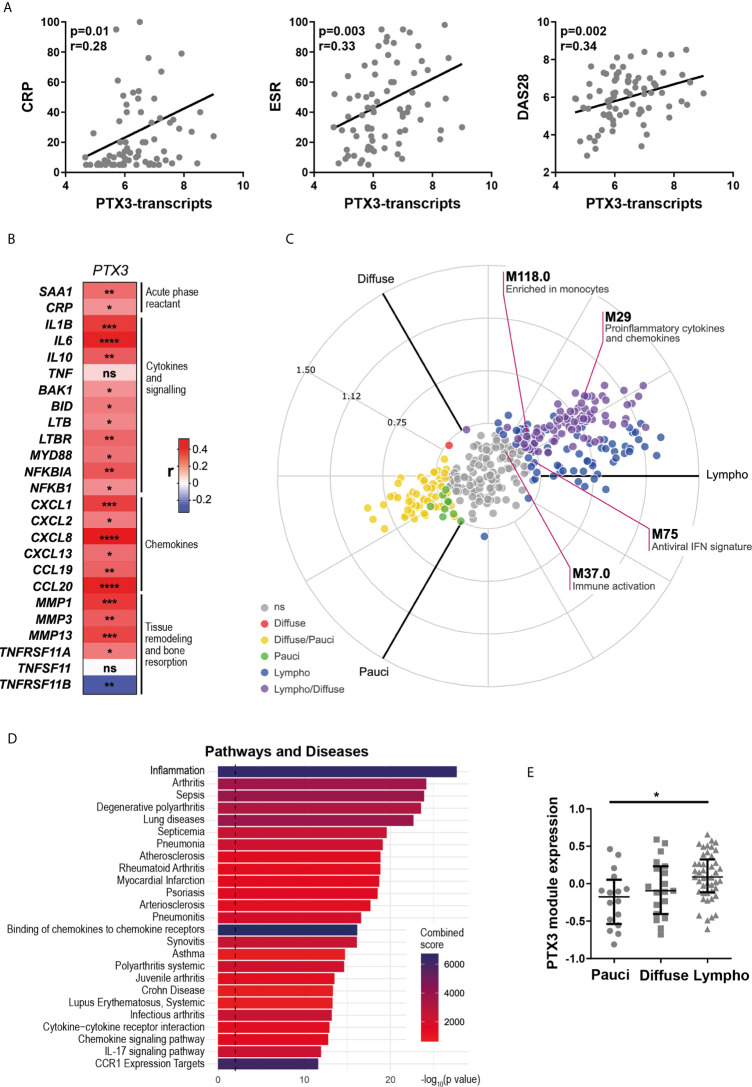
Synovial *PTX3* gene expression correlates with disease activity and local inflammation. **(A)** Correlation between synovial transcript levels of *PTX3* at baseline assessed by bulk RNA-seq and CRP, ESR and DAS28. **(B)** Heatmap representing the correlation between *PTX3* synovial transcript levels at baseline and acute phase reactants, selected chemokines, cytokines and related signaling pathways mediators involved in inflammation, tissue remodeling and bone resorption. The red/blue scale represents the spearman r coefficient. *p < 0.05, **p < 0.01, ***p < 0.001, ****p < 0.0001. **(A, B)** p-values and r coefficients were calculated using Pearson correlation test. **(C)** 2D polar plot of transcript modules containing *PTX3* in synovial tissue presenting a lympho-myeloid (lympho), diffuse-myeloid (Diffuse), and pauci-immune fibroid (Pauci) pathotypes (4). Different colors show pairwise comparisons between the three pathotypes: upregulation in one group only (Diffuse: red, Pauci: green and Lympho: blue) or in two groups (Diffuse/Pauci: yellow, Lympho/Diffuse: purple). **(D)** Pathways and diseases gene set enrichment analysis of the PTX3 module, composed of the 30 genes encoding for the protein integrated in the PTX3 string network, performed using the R interface to the EnrichR database (https://CRAN.R-project.org/package=enrichR). **(E)** PTX3 module expression in RA synovium classified as pauci-immune (n = 16), diffuse-myeloid (n = 20) and lympho-myeloid (n = 43). Data are represented as median +/- interquartile range. * = p < 0.05, as assessed by the Kruskal–Wallis test with Dunn’s post-test; ns, non-significant.

Here, we assessed the relationship between synovial *PTX3* expression, clinical disease activity and local inflammation. At baseline, RNA-seq analysis revealed that transcript levels of *PTX3* in the synovial tissue, similarly to what was observed in the peripheral blood (**Figure 1D**), strongly correlated with disease activity, assessed by ESR and circulating CRP concentrations and DAS28 (**Figure 2A**). In addition, synovial *PTX3* mRNA also correlated with the gene expression of other acute phase reactants, such as *CRP* or serum amyloid A (*SAA*), selected chemokines, cytokines and molecules involved in their signaling pathways, as well as mediators involved in promoting tissue remodeling and bone resorption, such as matrix metalloproteinases (*MMP*) or Receptor Activator of NF-κB (RANK, gene *TNFSFR11A*) (**Figure 2B**). Conversely, we observed a negative correlation between *PTX3* transcript expression and *TNFSFR11B*, the gene encoding for Osteoprotegerin (OPG), decoy receptor of RANK ligand (RANKL) and negative regulator of osteoclastogenesis. Of note, although the correlation between *PTX3* and *TNF* expression was not significant, several mediators involved in the TNF signaling cascade and cell death, such as BCL2 Antagonist/Killer 1 (*BAK1*), BH3 Interacting Domain Death Agonist (*BID*), Lymphotoxin Beta (*LTB*) and LTB receptor (*LTBR*) were positively and significantly associated with *PTX3* expression (**Figure 2B**).

We then applied blood transcript modules to the RA synovium RNA-seq dataset, as described in (4) (and see https://peac.hpc.qmul.ac.uk/). *PTX3* was present in four different modules, “pro-inflammatory cytokines and chemokines” (M29), “enriched in monocytes” (M118.0), “immune activation” (M37.0) and “antiviral IFN signature” (M75) (29) (**Figure 2C**). Importantly, 3 out of 4 of these modules were significantly upregulated in synovial tissue presenting lympho-myeloid and diffuse-myeloid pathotypes. In addition, a similar pattern of expression was observed using synovium modules, defined by weighted gene correlation network analysis (WGCNA) (4), in which *PTX3* was present in the “M1 macrophages, chemokines and cytokines” module (SC43) (**Supplementary Figure 2A**, and see https://peac.hpc.qmul.ac.uk/). Accordingly, a direct comparison of synovial *PTX3* transcript expression in the three pathotypes highlighted a higher expression of *PTX3* in the lympho-myeloid compared to the pauci-immune pathotype while the correlation was not obvious with the Krenn synovitis score (**Supplementary Figures 2B, C**). String network analysis identified 30 relevant known and predicted PTX3 physical and functional partners (**Supplementary Figure 2D**). We used these 30 molecules to define a PTX3 gene module. Using GSEA, we observed that this module was particularly highly associated with the development of inflammation, arthritis, and sepsis (**Figure 2D**) and with relevant ontology pathways, such as neutrophil-mediated immunity, cytokine signaling pathways or regulation of leukocytes migration (**Supplementary Figure 2E**). The enrichment of this PTX3 module associated with the presence of a lympho-myeloid pathotype (**Figure 2E**), as observed at the single PTX3 gene expression level, which prompted us to further investigate how PTX3 protein expression related to synovial histopathology and to characterize synovial PTX3-producing cells.

### Synovial PTX3 Expression Associates With Immune Cell Infiltration and the Presence of Ectopic Lymphoid Structures

**Figure 3:**
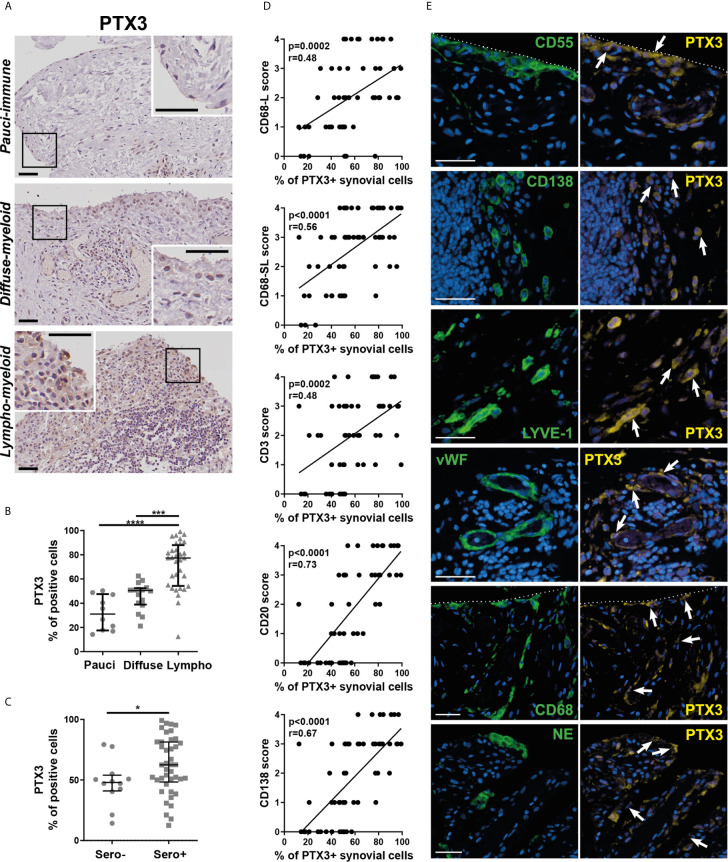
Synovial PTX3 expression associates with immune cell infiltration and the presence of ectopic lymphoid structure. **(A)** Sections of RA synovium were stained for PTX3. Representative images are shown for each pathotype (pauci-immune, diffuse-myeloid and lympho-myeloid). Scale bar = 40μm. **(B)** Digital image analysis was performed on RA synovium sections (n = 58) classified as pauci-immune (n = 10), diffuse-myeloid (n = 16) and lympho-myeloid (n=32). Synovial PTX3+ cells quantification was determined using QuPath software (28). ***p < 0.001, ****p<0.0001, as assessed by the Kruskal–Wallis test with Dunn’s post-test. **(C)** % of PTX3 positive cells in rheumatoid factor (RF)/cyclic citrullinated peptide (CCP) seropositive/negative patients. *p<0.05, as assessed by the Mann Whitney test. Data are represented as median +/- interquartile range. **(D)** Correlation between the percentage of PTX3 positive cells and the semi-quantitative scores for inflammatory cells markers (CD3-T cells; CD20-B cells; CD138-plasma cells; CD68L-macrophages of the synovium lining layer; CD68SL-macrophages of the sublining layer) (2, 3). p-values were calculated using Pearson correlation test. **(E)** Double immunostaining of PTX3 (yellow) with CD55, CD138, LYVE-1, vWF, CD68 and NE (green) in the synovium of RA patients. Nuclei were counterstained with DAPI (blue). White arrows indicate double-positive cells. Representative images are shown. Scale bar = 40μm.

To understand the relationship between PTX3 synovial expression and specific histopathological features of the tissue, we performed PTX3 IHC staining and positive cell quantification. Representative pictures of the staining in RA synovial tissue, classified as lympho-myeloid, diffuse myeloid or pauci-immune, are shown in **Figure 3A**. As observed at the transcript level, the percentage of PTX3-positive cells was significantly higher in synovial tissue presenting a lympho-myeloid pathotype, characterized by the presence of ELS, compared to diffuse-myeloid and pauci-immune pathotypes (**Figure 3B**). Next, we investigated whether, in line with its higher expression in the lympho-myeloid tissue, PTX3 protein expression was linked to the presence of RA auto-antibodies, which have been reported to associate with ELS-positive tissues (3). We showed a higher expression of synovial PTX3-positive cells in RF/CCP seropositive compared to seronegative patients (**Figure 3C**). In addition, in keeping with the higher expression of PTX3 in the lympho-myeloid pathotype, the percentage of PTX3 positive cells strongly correlated with synovial infiltration of CD3+ T cells, CD20+ B cells, CD138+ plasma cells and lining and sublining CD68+ macrophages (**Figure 3D**) and the total Krenn score (**Supplementary Figure 3A**). Double immunofluorescence staining confirmed that PTX3 was expressed by a large variety of synovial cells, mainly by CD55+ and TE7+ fibroblasts, CD138+ plasma cells, LYVE-1+ endothelial lymphatic cells, vWF+ endothelial cells, lining and sublining CD68+ macrophages, and NE+ neutrophils (**Figure 3E** and **Supplementary Figure 3B**). These results are in accordance with previously RNA-seq published data that highlighted the expression of PTX3 in both fibroblast and monocyte populations and a higher expression between monocytes sorted from leukocyte-rich and leukocyte-poor synovial tissue (30) (**Supplementary Figure 3C**, and see https://immunogenomics.io/ampra/).

Importantly, although we noticed a non-linear correlation between synovial PTX3 transcripts and protein expression (**Supplementary Figure 4**), the strong association between histopathology, and in particular the presence of a lympho-myeloid pathotype, and a high PTX3 expression was observed at both transcript and protein levels.

### Synovial PTX3 Expression Is Significantly Decreased After cs-DMARD Treatment but Independently of Clinical Response

**Figure 4:**
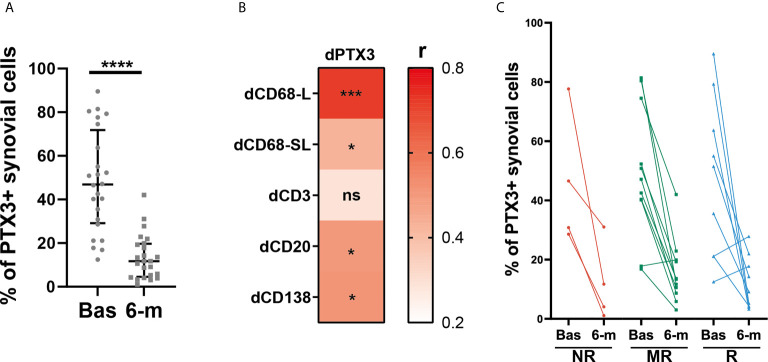
Synovial PTX3 expression is significantly decreased after cs-DMARD treatment but independently of clinical response. **(A)** % of PTX3 positive synovial cells in paired patients at baseline and at 6-months after cs-DMARD treatment. Data are represented as median +/- interquartile range. ****p < 0.00001, as assessed by the Wilcoxon test. **(B)** Heatmap representing the correlation between dPTX3 synovial expression (calculated as the difference of expression between baseline and 6-months) and difference between semi-quantitative scores for inflammatory cells markers at baseline and at 6-months (dCD3, dCD20, dCD138, dCD68L, dCD68SL). The red scale represents the spearman r coefficient. *p < 0.05, ***p < 0.001, ns, non-significant. p-values and r coefficients were calculated using Pearson correlation test. **(C)** % of PTX3 positive synovial cells at baseline (Bas) and at 6-months (6-m) in non-responders (NR, red lines), moderate responders (MR, green lines) and good responders (R, blue lines) patients to cs-DMARD treatment.

To further characterize the effects of cs-DMARD treatments on joint inflammation, a total of 24 synovial tissue biopsies collected at 6-months post-cs-DMARD treatment were stained for PTX3. Conversely to what was observed in serum, where PTX3 levels remained high at 6-months (**Figure 1B**), as a result of global decreased synovial cellularity, paired analysis revealed that the percentage of synovial positive PTX3 cells was significantly decreased following csDMARD treatment (**Figure 4A**). Indeed, in line with the association between PTX3 expression and the immune synovial infiltrate observed at baseline, decreased PTX3 levels were significantly associated with the reduction of semi-quantitative scores for inflammatory cells markers, in particular macrophage and B/plasma cell infiltration (**Figure 4B**). As observed at the peripheral level (**Figure 1G**), baseline and 6-month PTX3 synovial expression levels did not distinguish patients who responded from those who were resistant to cs-DMARDs (**Figure 4C**). Importantly, both at peripheral and local levels, the baseline level of PTX3 was not predictive of specific response status (**Figures 1G** and **4C**).

## Discussion

Here, for the first time, we analyzed PTX3 expression profiles in a cohort of early and treatment-naïve RA patients at baseline, and 6-months after cs-DMARD treatment. As previously described, we confirmed that RA patients presented a higher concentration of circulating PTX3 compared to healthy donors (13–15) and that PTX3 levels positively correlated with CRP (16). In addition, we showed that, at the onset of the disease prior to any treatment modification, circulating PTX3 levels also correlated with ESR, DAS28 and the count of swollen and tender joints, therefore being a strong marker of disease activity. However, serum PTX3 level did not differentiate patients presenting distinct synovial pathotypes, while, for example higher systemic levels of CRP were found in patients with a lympho-myeloid pathotype compared to other pathotypes, in line with a previous report from our group (3).

We also found that PTX3 serum levels were not reduced after treatment in paired samples, with 6-months values being proportional to baseline values and independent of a low/high clinical activity or erosion status at baseline. These results are in line with previously published evidence demonstrating that PTX3 serum levels are not significantly affected by various treatments for inflammatory arthritis, either cs-DMARDs (methotrexate) or anti-TNF or a combination of both, at different timepoints (6 weeks or 6 months) in a cohort including patients with RA, psoriatic arthritis and spondylarthritis (31). As previously reported by our group, steroid treatment might have opposite effects on the release of PTX3 by different cell types involved in the same inflammatory microenvironment, PTX3 expression being for example inhibited in myeloid cells and upregulated in fibroblasts and endothelial cells (32), illustrating the complexity of PTX3 production balance in response to therapies. Also, as reported by Satomura and colleagues, PTX3 expression is enhanced by other acute-phase reactants, such as SAA produced by hepatocytes, independently of treatment influence (33). Accordingly, we observed a positive correlation between *SAA* and *PTX3* synovial transcripts abundance in our cohort. The maintenance of high peripheral levels of PTX3, despite cs-DMARD treatment, might therefore participate in the perpetuation of systemic inflammation in RA and promote the occurrence of disease flares. Importantly, as synovitis and overall local joint inflammation is considered a hallmark of RA, we provided, for the first time, complementary analysis in matched blood and synovial samples from patients with RA at the same timepoint before and after treatments. We demonstrated that, as a result of the global decreased synovial cellularity, local expression of PTX3 significantly diminished following cs-DMARD treatment. Cs-DMARD are indeed known to influence the phenotypes and functions of several synovial immune cells, including for example macrophages (34) or neutrophils (35), which both represents major sources of synovial PTX3. The decreased PTX3 expression following cs-DMARD treatments was, however, independent of the response status, and deciphering the exact mechanisms by which cs-DMARD influence PTX3 production in the complex RA local and peripheral environments still requires further investigations. Here, we confirmed that PTX3 cannot be considered a predictive marker of response to cs-DMARDs either in the circulation or at the local disease tissue level.

Post-transcriptional modifications of PTX3 protein have been shown to finely regulate PTX3 functions in native immunity and inflammation (36). PTX3 is characterized by a N-glycosylation site in its C-terminal domain and PTX3 glycosylation pattern changes depending on cell types and inducing stimuli, which contribute to modulate PTX3 interactions with its ligands (e.g., complement components, extracellular matrix proteins) to tune related functions, including the recruitment of inflammatory cells to the site of inflammation (6, 37). Although we observed that synovial transcript and protein levels did not significantly correlate, suggesting that local post-transcriptional regulations may affect PTX3 expression in the joint, a strong association between PTX3 and the presence of specific pathotypes was maintained at both levels.

Notably, PTX3 protein was significantly more expressed in synovial tissues displaying a lympho-myeloid pathotype, characterized by the presence of ELS, compared to diffuse-myeloid or pauci-immune pathotypes. Accordingly, PTX3 synovial expression positively correlated with the levels of CXCL- and CCL- chemokines involved in ELS formation and maintenance, such as CXCL13 or CCL19 (38). In keeping with the high levels of PTX3 in highly infiltrated synovial tissues, we observed a significant correlation between PTX3 synovial expression and either the total Krenn score or semi-quantitative assessment of lining and sublining CD68+ macrophages, CD3+ T cells, CD20+ B cells and CD138+ plasma cells infiltration. PTX3 was expressed in numerous infiltrating and resident cell types activated during synovial inflammation, such as vascular and lymphatic endothelial cells, fibroblasts, plasma cells, macrophages and neutrophils. Importantly, we demonstrated for the first time that a high PTX3 synovial tissue expression was associated with seropositivity for RF/anti-CCP autoantibodies, as highlighted by Weitoft and colleagues in the synovial fluid of RA patients (21). Thus, synovial PTX3 might act as a bridge between innate and adaptive immunity, as previously reported in other tissues. For example, in the spleen PTX3 produced by activated neutrophils can bind B cells in the marginal zone, promote homeostatic production of IgM and class-switched IgG antibodies to microbial capsular polysaccharides and enhance the immunogenic response to blood-borne bacterial infection (39). Similarly, PTX3 has been shown to bind microbial moieties from *Neisseria meningitidis*, act as an endogenous adjuvant of response and amplify the induction of an effective adaptive antibody response (40). Recently, the presence of anti-PTX3 autoantibodies has been described in RA and shown to positively correlate with PTX3 serum levels, disease activity and circulating levels of pro-inflammatory cytokines, such as IL-6 or IL-1β (41), further confirming PTX3 immunogenicity in the RA context.

PTX3 roles within the arthritic tissue is still being debated. Our RNA-seq analysis confirmed a strong association between PTX3 expression in the synovial tissue, disease activity and the presence of cytokines and chemokines driving local inflammation, tissue remodeling and bone erosions. Although these correlations are not proof of a causal link between PTX3 expression and disease features, it provides important insights into the relationship between PTX3 synovial levels and the inflammatory environment associated with synovial histopathology. Importantly, these results are consistent with protein data. In addition, the expression of the 30 genes included in our PTX3 module are highly associated with the development of inflammation, arthritis, and sepsis. Of note, PTX3 has been described by our group and others as a functional player and relevant biomarker in sepsis (42). Recently, Wu and colleagues (43) also demonstrated an important role of IL-6 in driving PTX3/C1q-mediated pyroptosis and inflammasome activation in RA. In addition, Garcia and colleagues demonstrated that MMP8 deficiency leads to an increased disease severity, accompanied by an increase expression of IL1B and PTX3 in the joints (44). While the participation of PTX3 in driving joint inflammation is now well documented, its contribution to bone remodeling is more controversial and still requires further investigations (45). For example, a recent report highlighted a protective role of PTX3 through the inhibition of fibroblast growth factor 2 (FGF-2) associated osteoclastogenesis in the collagen-induced arthritis model (46). In this model, the injection of recombinant PTX3 reduces inflammatory scores and bone erosions. In addition, the binding of PTX3 to FGF-2 could reverse the inhibitory effect of this growth factor on osteoblastogenesis, therefore confirming its influence on physiological and pathological bone remodeling (47). In contrast, and aligned with recent studies (20, 48) demonstrating an association between PTX3 circulating levels and the progression of joint damage in RA, osteoporosis and osteoarthritis bone phenotype, we confirmed that PTX3 serum levels measured at the onset of RA and prior to treatment modification correlates with radiographic damage observed at 12 months follow-up. In addition, in line with our previous report that patients presenting with a lympho-myeloid pathotype were more likely to develop joint damage progression, compared with the diffuse-myeloid and pauci-immune-fibroid pathotypes (3), here we observed that PTX3 expression was significantly elevated in the lympho-myeloid pathotype. This work therefore provides a better characterization of the relationship of PTX3 in the inflamed RA synovial tissue, and further improves our understanding of key pathways associated with the development of pathotypes in individual patients and joint erosions.

Hence, the measurement of PTX3 circulatory levels provides relevant information about disease activity and evolution of radiographic damage and its expression within the synovium also significantly associates with deleterious tissue infiltration. Noteworthy, PTX3 expression has also been associated with the development of RA comorbidities such as vasculitis (49, 50) or coronary artery disease (51, 52). Therefore, serum and synovial levels of PTX3 in RA provide additional insights into the disease pathogenesis and help anticipating increased risks of complications.

## Data Availability Statement

The datasets presented in this study can be found in online repositories. The names of the repository/repositories and accession number(s) can be found below: ArrayExpress [accession: E-MTAB-6141].

## Ethics Statement

The studies involving human participants were reviewed and approved by the National Research Ethics Service Committee London Dulwich (REC 05/Q0703/198). The patients/participants provided their written informed consent to participate in this study.

## Author Contributions

All authors have significantly contributed to enable the delivery of the present manuscript, either by performing the experiments, analyzing the results, recruiting patients, writing or critically revising the manuscript. All authors have read and approved it for publication. All authors contributed to the article and approved the submitted version.

## Funding

This work was supported by departmental funds (Queen Mary University of London, Centre for Experimental Medicine and Rheumatology and Department of Inflammation and Immunology, Humanitas Clinical and Research Center). AN is funded by Versus Arthritis Clinical Lectureship in Experimental Medicine and Rheumatology [grant number 21890] and M-AB by the Fondation pour la recherche médicale [grant number ARF202004011786]. MRC support has been obtained for the development of the PEAC cohort [grant number 36661] and Versus Arthritis for infrastructure support (Experimental Arthritis Treatment Centre [grant number 20022]). AM and BB are grateful to Fondazione Cariplo [contract number 2015-0564], the European Research Council (ERC, [grant number 669415]) and the Italian Spacial Agency (ASI MARS-PRE Project, [grant number DC-VUM-2017-006]) for financial support.

## Conflict of Interest

AM and BB are inventors of patents on pentraxin-3 and obtain royalties on related reagents. The authors declare that there are no other commercial or financial relationships that could be construed as a potential conflict of interest.
