# Supplementary material for: Circulating and Synovial Pentraxin-3 (PTX3) Expression Levels Correlate With Rheumatoid Arthritis Severity and Tissue Infiltration Independently of Conventional Treatments Response
Source: Front Immunol. 2021 Jun 25;12:686795. doi: 10.3389/fimmu.2021.686795 (PMC8267520; doi:10.3389/fimmu.2021.686795)

Supplementary Material

**Supplementary Figure 1**: The change in serum levels of PTX3 between baseline and 6-months post-treatment is independent of clinical disease severity at baseline and treatment response status. **(A)** dPTX3 serum levels between baseline and 6-months post-treatment in patients presenting a low/high CRP, ESR or DAS28, erosions or no erosion at baseline. **(B)** dPTX3 serum levels between baseline and 6-months post-treatment in non-responders (NR, red dots), moderate responders (MR, green dots) and good responders (R, blue dots) to csDMARDs treatments. **(A, B)** Data are represented as median +/- interquartile range.


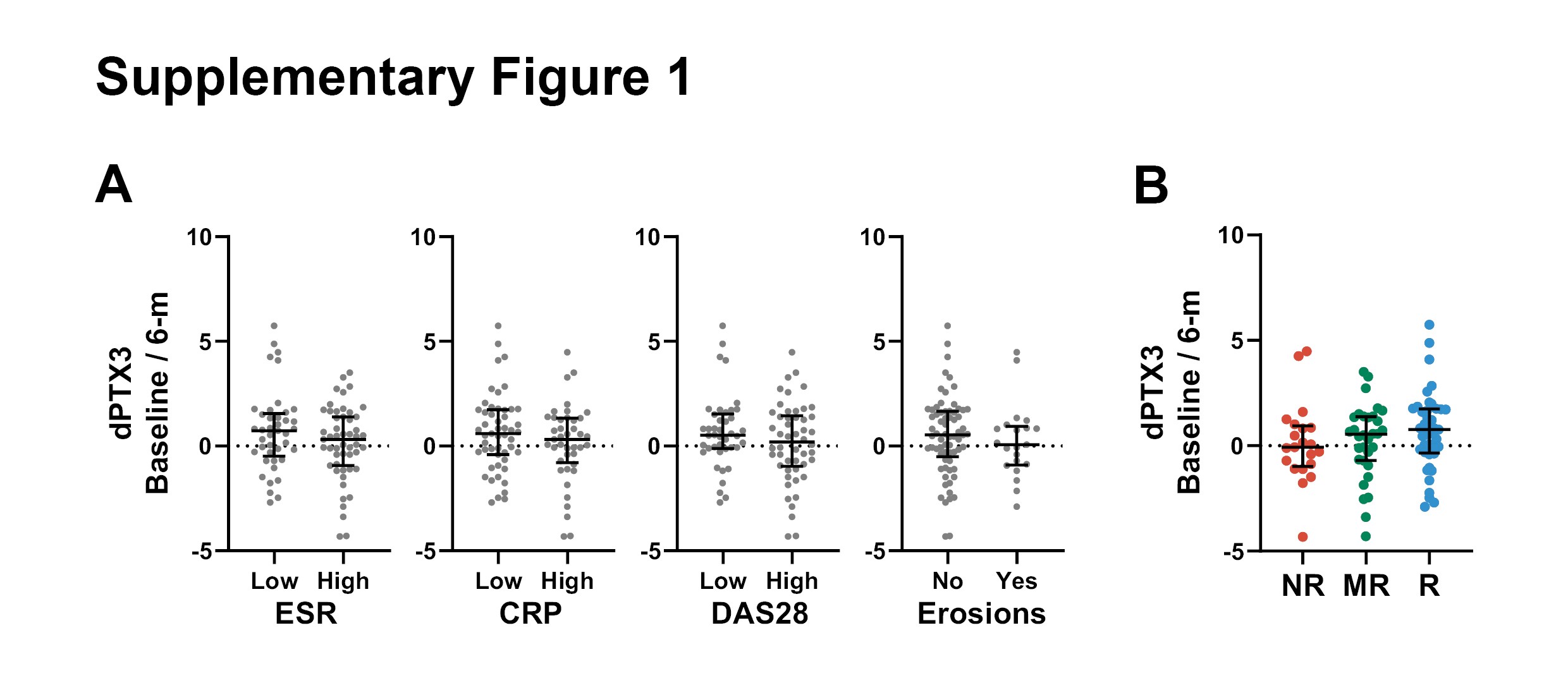


**Supplementary Table 1**: RNA-seq (n=79) and IHC (n=58) subpopulations have similar baseline clinical characteristics compared to the total population analysed by ELISA (n=119). p-values were calculated by comparing the total population and both subgroups using Kruskal Wallis or Fisher's exact/Chi-squared test as appropriate. n, number; IQR, interquartile range; RF, rheumatoid factor; CCP, cyclic citrullinated peptide; ESR, erythrocyte sedimentation rate; CRP, C-Reactive Protein; PTX3, pentraxin-3; TJC, tender joints count; SJC, swollen joints count; VAS, Visual Analogue Scale (0-100); HAQ, Health Assessment Questionnaire; DAS, Disease Activity Score; DMARDs, Disease Modifying Anti-Rheumatic Drugs; ns, non-significant.


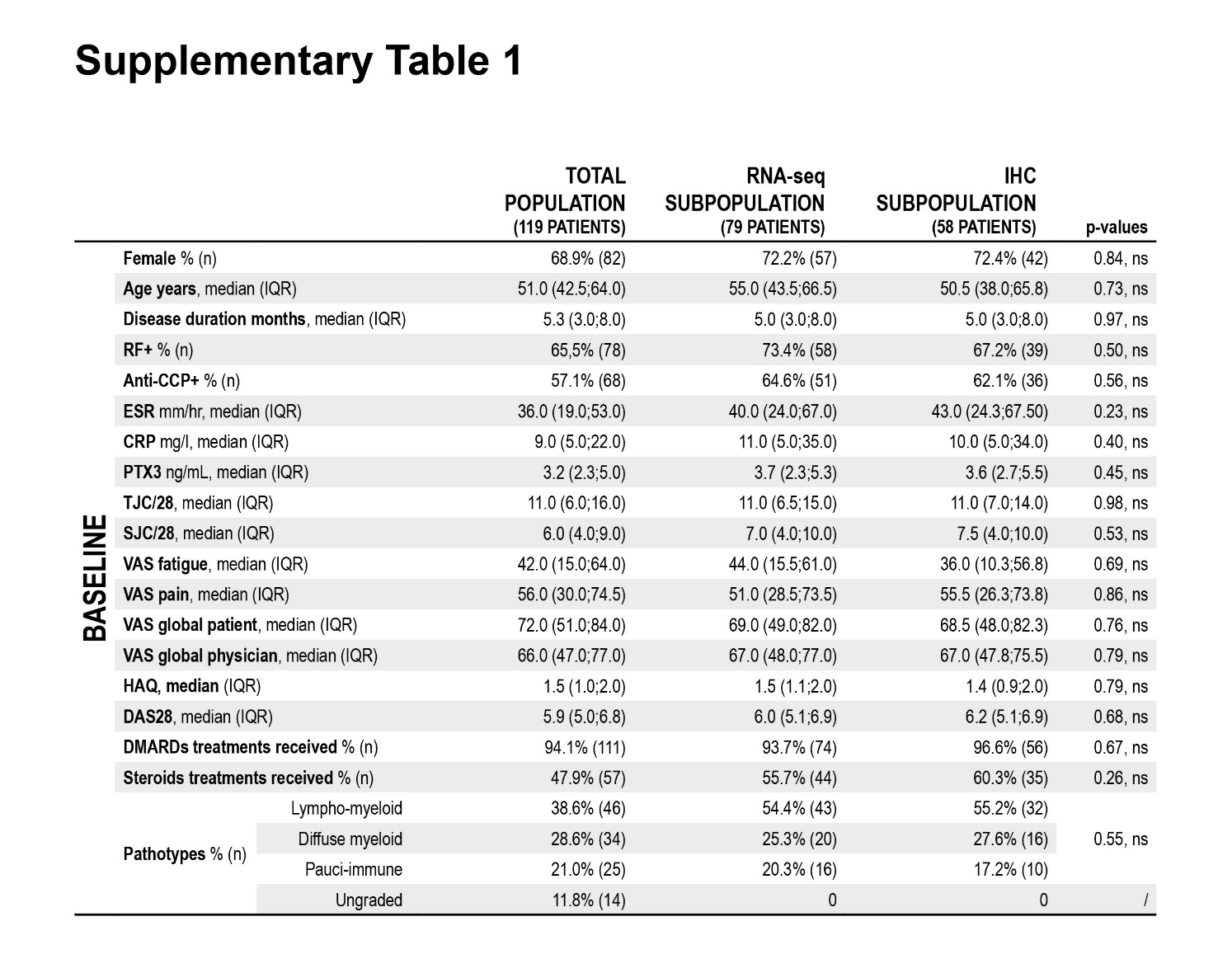


**Supplementary Figure 2**: Synovial PTX3 gene expression associates with histopathology. **(A)** 2D polar plot of WGCNA transcript modules containing PTX3 in synovial tissue presenting a lympho-myeloid (Lympho), diffuse-myeloid (Diffuse), and pauci-immune fibroid (Pauci) pathotypes (Lewis et al, 2019). Different colours show pairwise comparisons between the three pathotypes: upregulation in one group only (Diffuse: red, Pauci: green and Lympho: blue) or in two groups (Diffuse/Pauci: yellow, Lympho/Diffuse: purple). **(B)** PTX3 transcript expression in RA synovium classified as pauci-immune (n=16), diffuse-myeloid (n=20) and lympho-myeloid (n=43). Data are represented as median +/- interquartile range. * = p<0.05, as assessed by the Kruskal–Wallis test with Dunn's post-test. **(C)** Correlation between the PTX3 transcript expression and synovitis Krenn score. p-values were calculated using Pearson correlation test. **(D)** String functional PTX3 protein network (<https://string-db.org/>). **(E)** Ontologies gene set enrichment analysis of the PTX3 module, composed of the 30 genes encoding for the protein integrated in the PTX3 string network, performed using the R interface to the EnrichR database.


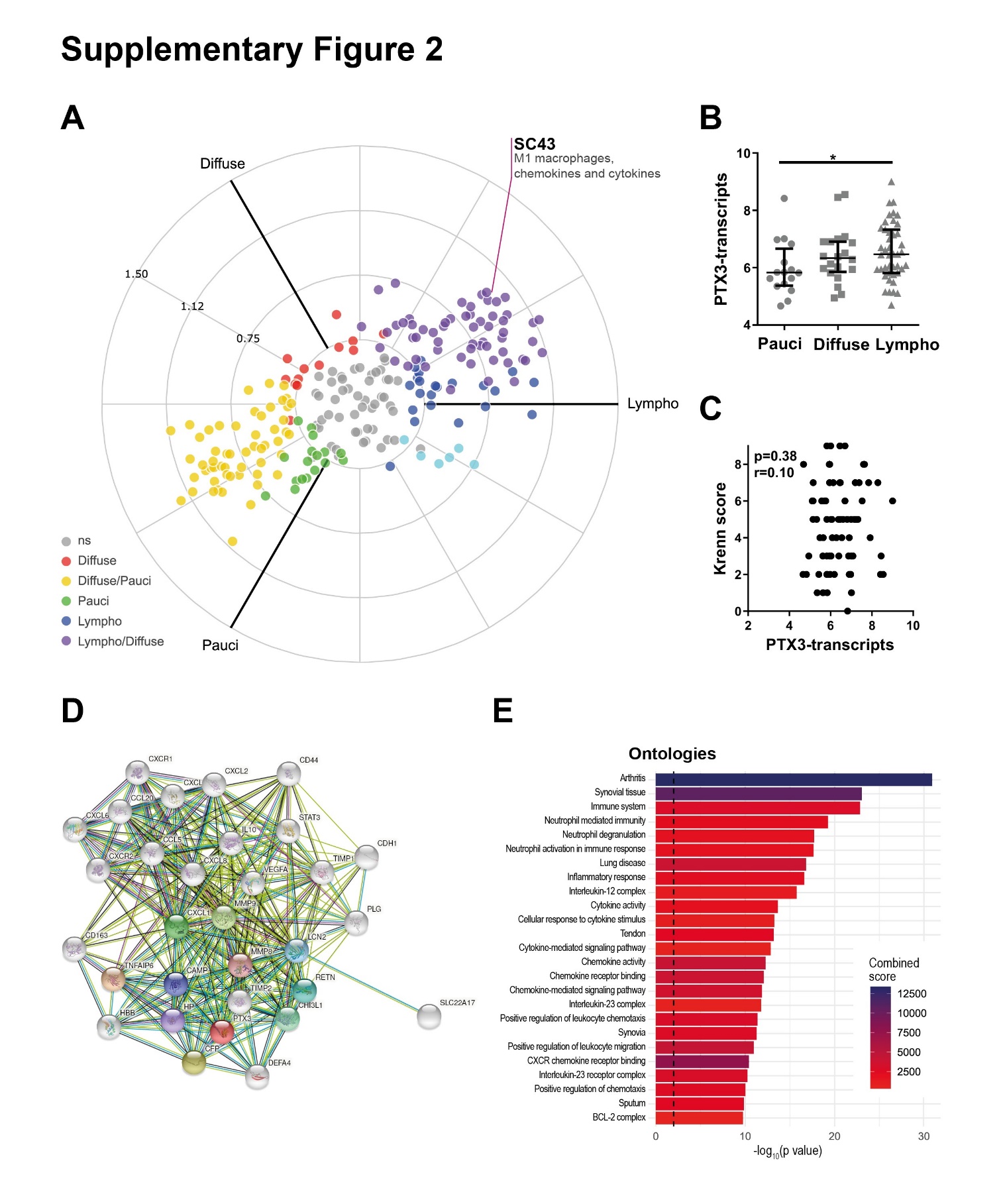


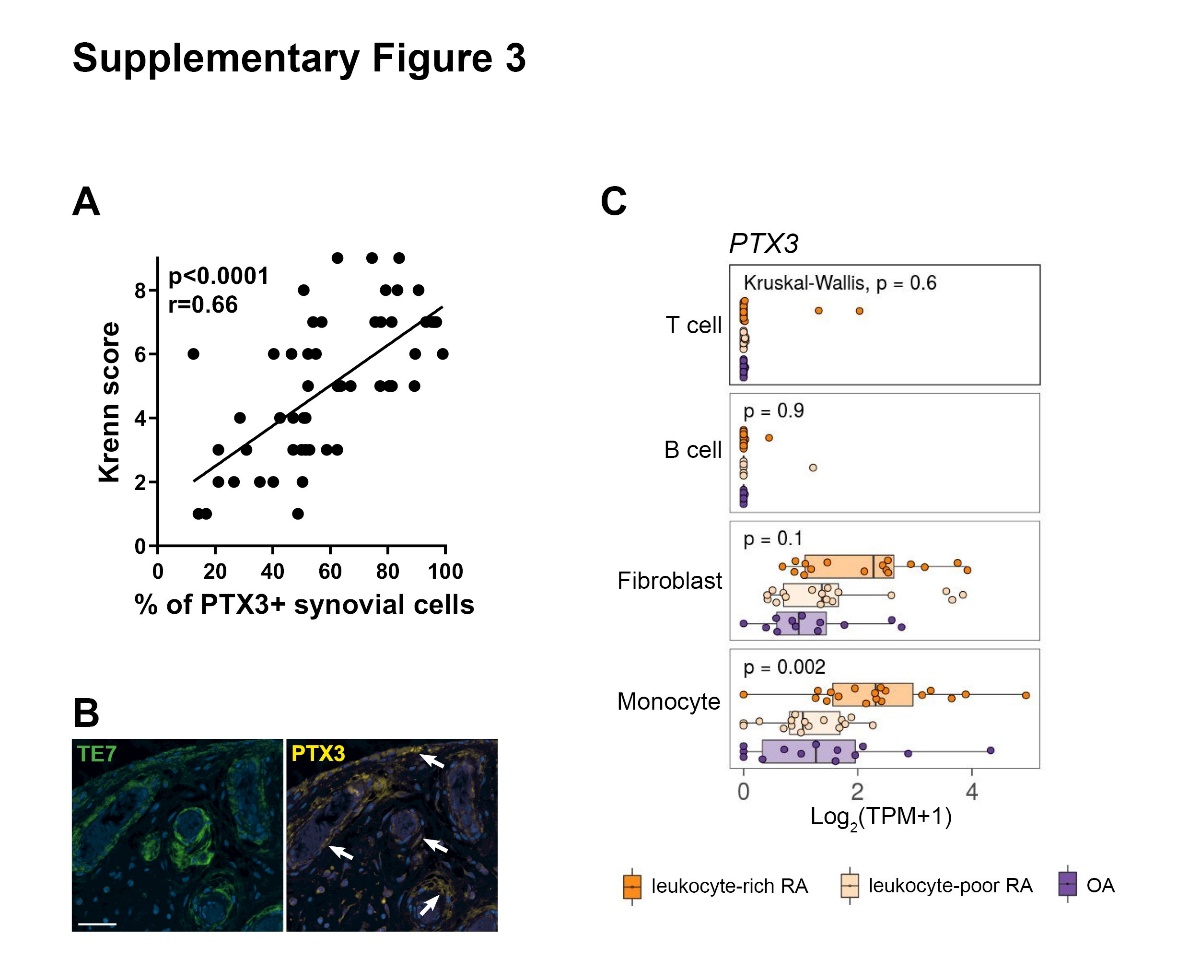
**Supplementary Figure 3**: Sources of synovial PTX3. **(A)** Correlation between the percentage of PTX3 positive cells and the Krenn score. p-values were calculated using Pearson correlation test. **(B)** Double immunostaining of PTX3 (yellow) with TE7 (green) in the synovium of RA patients. Nuclei were counterstained with DAPI (blue). White arrows indicate double-positive cells. Representative images are shown. Scale bar = 40μm. **(C)** Bulk RNA-seq data downloaded from <https://immunogenomics.io/ampra/> (Accelerating Medicines Partnership Rheumatoid Arthritis and Systemic Lupus Erythematosus (AMP RA/SLE) Consortium et al., 2019) showing the expression of PTX3 in B cells, T cells, fibroblasts, and monocytes in leukocyte-rich, leukocyte-poor and osteoarthritis synovial tissue.

**Supplementary Figure 4**: Transcript, but not protein levels of synovial PTX3 correlate with PTX3 serum levels. **(A)** Correlation between serum levels of PTX3 and synovial transcript levels of PTX3 (*left panel*), % of PTX3 synovial positive cells and PTX3 synovial transcript levels (*middle panel*) and between PTX3 serum levels and % of PTX3 synovial positive cells (*right panel*). p-values and r coefficients were calculated using Pearson correlation test.


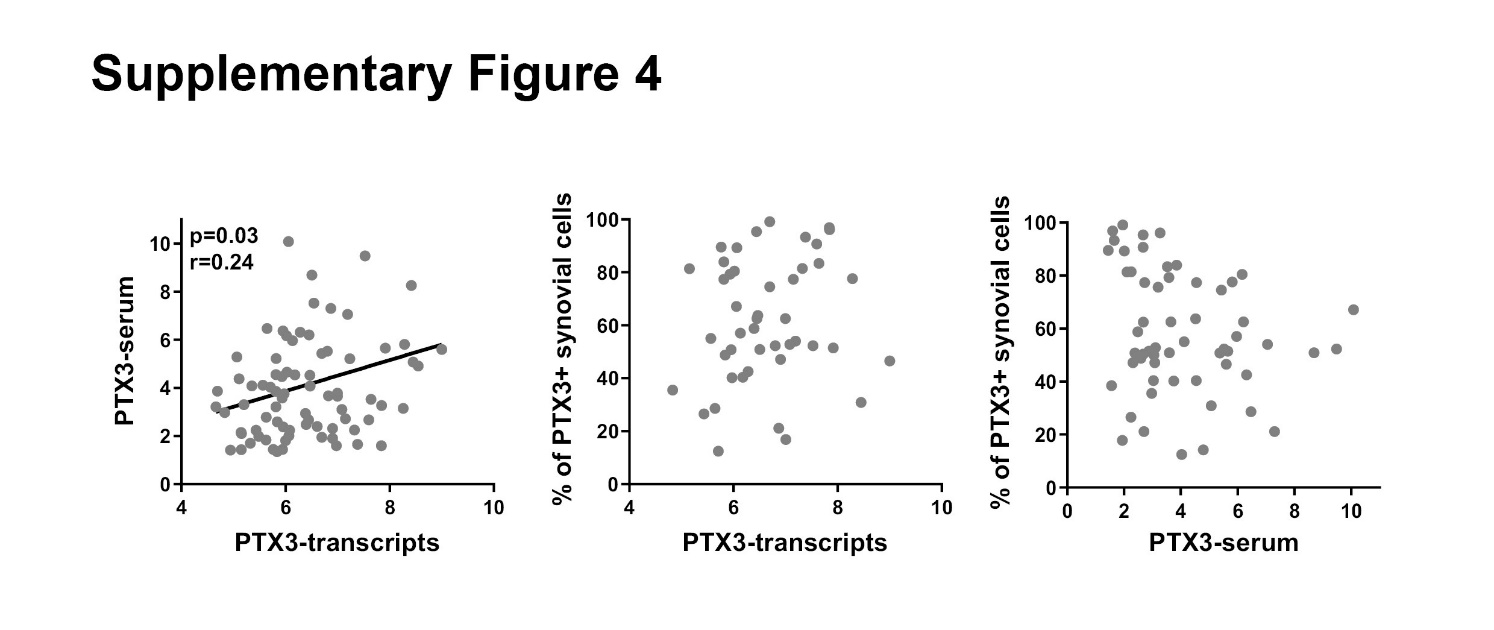

Supplement: Supplementary file 1 [file DataSheet_1.docx]
